# Expression and Functional Analysis of a Novel Group of Legume-specific WRKY and Exo70 Protein Variants from Soybean

**DOI:** 10.1038/srep32090

**Published:** 2016-08-30

**Authors:** Ze Wang, Panfeng Li, Yan Yang, Yingjun Chi, Baofang Fan, Zhixiang Chen

**Affiliations:** 1 Department of Horticulture, Zijingang Campus, 866 Yuhangtang Road, Zhejiang University, Hangzhou, 310058, China; 2 Department of Botany and Plant Pathology, 915 W. State Street, Purdue University, West Lafayette, IN 47907, USA

## Abstract

Legumes fix atmospheric nitrogen through symbiosis with microorganisms and contain special traits in nitrogen assimilation and associated processes. Recently, we have reported a novel WRKY-related protein (GmWRP1) and a new clade of Exo70 proteins (GmExo70J) from soybean with homologs found only in legumes. GmWRP1 and some of the GmExo70J proteins are localized to Golgi apparatus through a novel N-terminal transmembrane domain. Here, we report further analysis of expression and functions of the novel *GmWRP1* and *GmExo70J* genes. Promoter-GUS analysis in Arabidopsis revealed distinct tissue-specific expression patterns of the *GmExo70J* genes not only in vegetative but also in reproductive organs including mature tissues, where expression of previously characterized *Exo70* genes is usually absent. Furthermore, expression of some *GmExo70J* genes including *GmExo70J1*, *GmExo70J6* and *GmExo70J7* increases greatly in floral organ-supporting receptacles during the development and maturation of siliques, indicating a possible role in seed development. More importantly, suppression of *GmWRP1*, *GmExo70J7*, *GmExo70J8* and *GmExo70J9* expression in soybean using virus- or artificial microRNA-mediated gene silencing resulted in accelerated leaf senescence and reduced nodule formation. These results strongly suggest that legume-specific GmWRP1 and GmExo70J proteins play important roles not only in legume symbiosis but also in other processes critical for legume growth and development.

Legumes are important plants, second only to the grass family in economic and nutritional value. Legumes are capable of fixing atmospheric nitrogen through intimate symbiosis with soil bacteria collectively known as rhizobia. An estimated 17,000 to 19,000 legume species have been found in nature and many of them are important food plants including common bean (*Phaseolus vulgaris*), soybean (*Glycine max*) and pea (*Pisum sativum*) and important forage plants such as alfalfa (*Medicago sativa*) and clover (*Trifolium* spp)^1^. These grain and forage legumes are grown on about 15% of the world’s arable surface, providing about 33% of human’s dietary nitrogen, with up to 60% in developing countries^2^. There is also increasing interest in the use of legume as a source of biomass for biofuel production. Despite their importance, our understanding of many of the unique and special traits of legumes including the evolution of symbiotic nitrogen fixation and associated processes is still very limited. A better knowledge about the unique traits that define legumes, however, is required for development of new strategies to improve this important family of plants.

To date, genome sequence information from several legume species including soybean is available and has started to shed light on the diversity, evolution and molecular and genetic basis of unique and special traits of legumes. These sequenced legumes belong to the Papilionoideae, which appears to have undergone a whole- genome duplication event some 60 million years ago^3^. Soybean is an ancient polyploid with a second genome duplication that occurred at approximately 13 million years ago, followed by gene loss and diversification and chromosome rearrangements^4^. It has also been revealed that many of the genes and pathways in legumes including nodulation, isoflavonoid biosynthesis and vesicle trafficking of storage proteins evolved from those shared by other plant species^4^. Likewise, analysis of genes encoding transcription factors did not reveal a legume-specific family^5^. To develop specialized functions, therefore, legumes appear to have largely relied on diverting common gene families through changes in sequences of coding and regulatory regions, thereby altering their structures, expression and ultimately functions. Indeed, legumes have novel genes that have diverged greatly from their progenitors with new functions important for legume-specific traits. For example, a previous study using BLAST algorithms has identified putative legume-specific genes with no sequence homology, below a specified threshold, to sequences of nonlegumes^6^. Identification and functional analysis of legume-specific genes could provide novel insights into the genetic and molecular basis of hallmark legume traits.

During our recent analysis of the soybean WRKY transcription factor superfamily, we have reported a WRKY-related soybean protein (GmWRP1) that appears to have derived from the fusion of the N-terminal WRKY domain of a Group I WRKY protein with a novel N-terminal five-pass transmembrane (TM) domain^7^. Unlike typical WRKY proteins, GmWRP1 is unable to bind the TTGACC W box sequences and is exclusively targeted to Golgi complex through its N-terminal TM domain^7^. Similar Golgi-targeting TM domains are also identified in a number of soybean proteins including members of a subfamily of 12 Exo70J proteins^7^. Exo70 is a critical component of the exocyst complex consisting of Sec3, Sec5, Sec6, Sec8, Sec10, Sec15, Exo70 and Exo84^8^,^9^,^10^,^11^,^12^. The exocyst complex mediates the tethering of secretory vesicles at the plasma membrane for exocytosis and cell-surface expansion. Unlike other exocyst subunits, the genes for Exo70 have proliferated greatly in plants with 23 in Arabidopsis^13^,^14^. Plant Exo70 proteins have been divided into three clades (Exo70.1 to Exo70.3) and nine subclades (Exo70A to Exo70I)^13^,^14^. The soybean Exo70J subfamily is phylogenetically distinct from the nine subclades of Exo70 proteins previously identified from other plants^7^. Intriguingly, homologs of soybean GmWRP1 and the subfamily of GmEx070J proteins are identified only in legumes but not in other plant species^7^. Transient overexpression of some of the legume-specific GmExo70 proteins or the Golgi-targeting TM domain in tobacco leave drastically changed the subcellular structures labeled by a fluorescent Golgi marker^7^. We have proposed that the novel legume-specific *GmWRP1* and *GmExo70J* genes, which resulted from evolution of two highly expanded plant gene families, are involved in vesicle trafficking of biological molecules highly important for legumes^7^.

To characterize GmWRP1 and those GmExo70J proteins with a novel N-terminal TM domain, we have previously examined their expression in soybean using qRT-PCR^7^. The analysis revealed that expression of *GmWRP1* was detected at various levels in different tissues including leaves, stem, roots, flowers and pods. In leaves, the expression of GmWRP1 was low at young ages (1-week old), but increased steadily with increased leaf ages^7^. In the 9–10 weeks old leaves, the expression levels were 50–80 times higher than those in the 1-week old leaves^7^. Age-regulated expression was also observed with the seven GmExo70J proteins with the N-terminal TM domain^7^. Thus, the expression of the genes does not appear to be restricted to only those elongating and differentiating cells and the enhanced expression in older leaves would suggest age-promoted expression patterns.

Here, we report further analysis of expression and functions of the novel *GmWRP1* and *GmExo70J* genes. Promoter-GUS analysis of the 12 *GmExo70J* genes in Arabidopsis revealed distinct tissue-specific expression patterns in both vegetative and reproductive tissues including those mature organs and tissues where expression of *Exo70* genes belonging to other subfamilies is usually absent. Furthermore, expression of some *GmExo70J* genes suggests a possible role in vascular transport and seed development. For direct functional analysis, we have tried to suppress the expression of *GmWRP1* and *GmExo70J* genes in soybean using virus or artificial microRNA mediated gene silencing. The results from the silencing experiments strongly suggest that the legume-specific GmWRP1 and GmExo70J proteins play important roles in regulation of plant leaf senescence and root nodulation.

## Results

### Promoter activities of the *GmExo70J* genes in Arabidopsis

**Figure 1:**
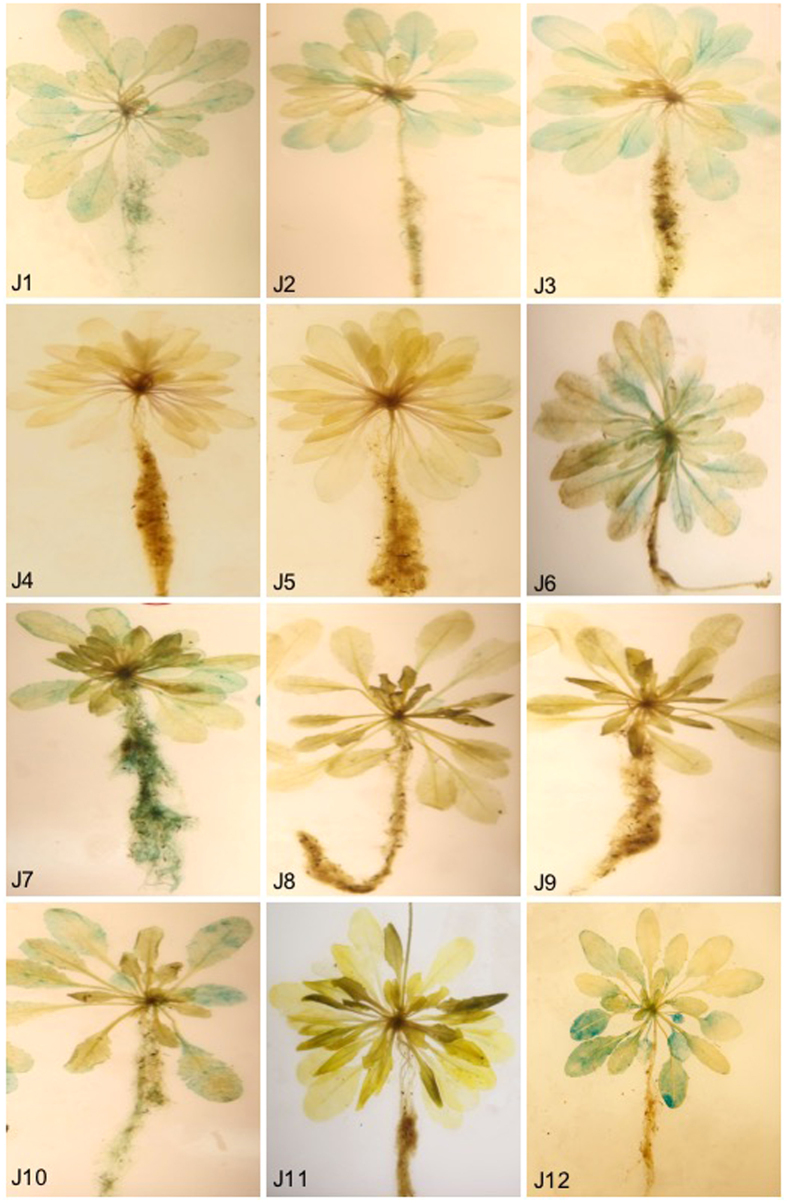
Histochemical analysis of *GmExo70J* promoter activities in the vegetative tissues of transgenic Arabidopsis plants. The promoter-GUS constructs for the 12 *GmExo70J* genes (J1 to J12) were introduced into wild-type Arabidopsis Col-0 plants. The promoter activities of 5-weeks old T2 progeny were histochemically analyzed.

The expression of Arabidopsis *Exo70* genes is tightly associated with exocytosis-active cells undergoing elongation and differentiation but is usually absent in mature organs or tissues^15^,^16^. To further characterize the expression of the 12 *GmExo70J* genes, we have analyzed their promoter activity of in Arabidopsis because of its ease for transformation. Expression of reporter genes driven by plant promoters in a heterologous system such as Arabidopsis is a widely used system that allows for fast and reliable analysis and dissection of plant promoter activity. Therefore, even though these genes are legume-specific, analysis of the promoter activities of these soybean genes in Arabidopsis could provide a reasonably reliable picture about their expression patterns of their native plants. For this purpose, we first isolated the ~1.5 kb genomic fragments upstream of the start codons of the 12 *GmExo70J* genes and placed them upstream of the *E. coli* reporter gene *uidA* encoding the GUS reporter enzyme in a plant transformation vector. The *GmExo70J::GUS* fusion constructs were introduced into Arabidopsis through *Agrobacterium*-mediated transformation and the T2 transgenic Arabidopsis plants containing the reporter gene constructs were subjected to histochemical staining for GUS activity. Figure 1 showed the activity profiles of the 12 *GmExo70J* gene promoters in the vegetative tissues of the 5-weeks old Arabidopsis plants. Based on the intensity of GUS staining, the promoter activities of the *GeExo70J* genes varied. The promoter activities of *GmExo70J1*, *GmExo70J2*, *GmExo70J3*, *GmExo70J6*, *GmExo70J7* and *GmExo70J10* were readily detected in shoots, particularly in the vascular tissues (Fig. 1). Promoter activities in roots were also detected for *GmExo70J1*, *GmExo70J2*, *GmExo70J10* and, at higher levels, *GmExo70J7* (Fig. 1). The other six *GmExo70J* genes had relatively low levels of promoter activities in the vegetative tissues (Fig. 1).

**Figure 2:**
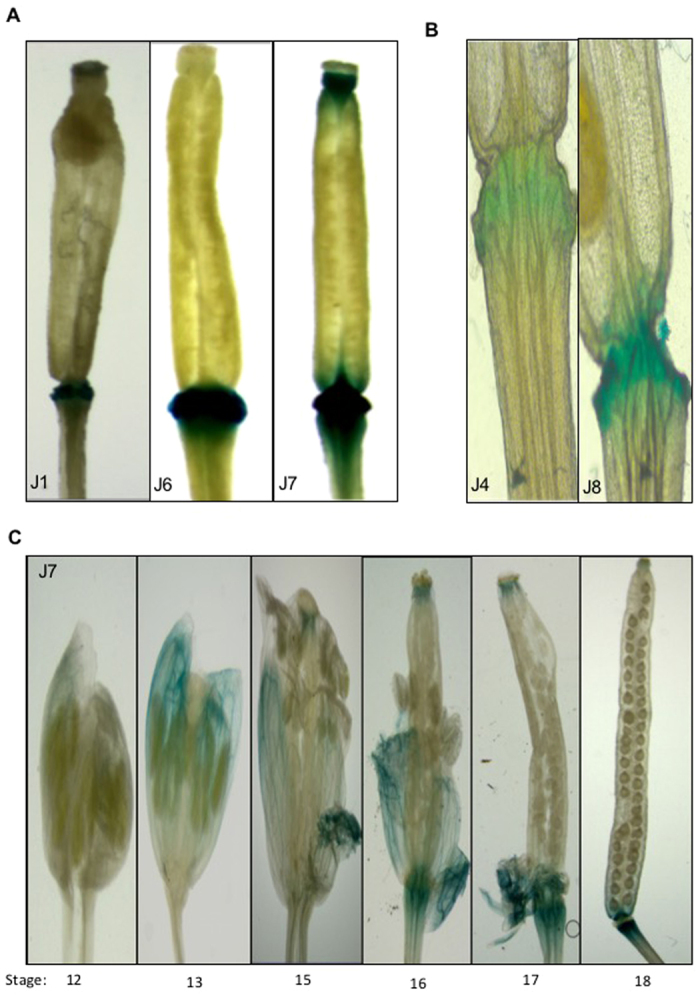
The promoter activity of *GmExo70J* genes in the floral organ-supporting receptacles of transgenic Arabidopsis. (**A**) Histochemical analysis of the promoter activities of *GmExo70J1* (J1), *GmExo70J6* (J6) and *GmExo70J7* (J7) in the floral organ-supporting receptacles of transgenic Arabidopsis flowers at stage 17. (**B**) Histochemical analysis of the promoter activities of *GmExo70J4* (J4) and *GmExo70J8* (J8) in the floral organ-supporting receptacles of transgenic Arabidopsis flowers at stage 17. (**C**) Histochemical analysis of the promoter activity of *GmExo70J7* (J7) in the floral organ-supporting receptacles of transgenic Arabidopsis flowers at different stages.

We also performed histochemical staining for GUS activity in the reproductive tissues of the transgenic Arabidopsis and revealed interesting activity patterns for some of these *GmExo70J* gene promoters. First, we found strong promoter activities of *GmExo70J1*, *GmExo70J6* and *GmExo70J7* in the floral organ-supporting receptacles (Fig. 2A). Promoter activities of *GmExo70J4* and *GmExo708* were also detected, albeit at lower levels, in the receptacles of transgenic Arabidopsis plants (Fig. 2B). Furthermore, the tissue-specific expression of these *GmExo70J* genes in the receptacles is highly developmentally regulated. Flower development can be divided into 20 stages from stage 1 when flower buttress arise to stage 20 when seeds fall^17^. As shown in Fig. 2C, no significant promoter activity of *GmExo70J7* was detected at stage 12 in closed young flower tissues. At stage 13, when flowers open, the promoter activity of *GmExo70J7* became detected in the sepal tissues but not in the receptacles (Fig. 2C). The promoter activity of *GmExo70J7* was first detected in the receptacles at stages 15 and 16 when stigma extended above long anthers and petals and sepals started withering (Fig. 2C). At stages 17 and 18 when all floral organs continued to fall during the development and maturation of siliques, the promoter activity of *GmExo70J7* increased substantially in the receptacles (Fig. 2C).

**Figure 3:**
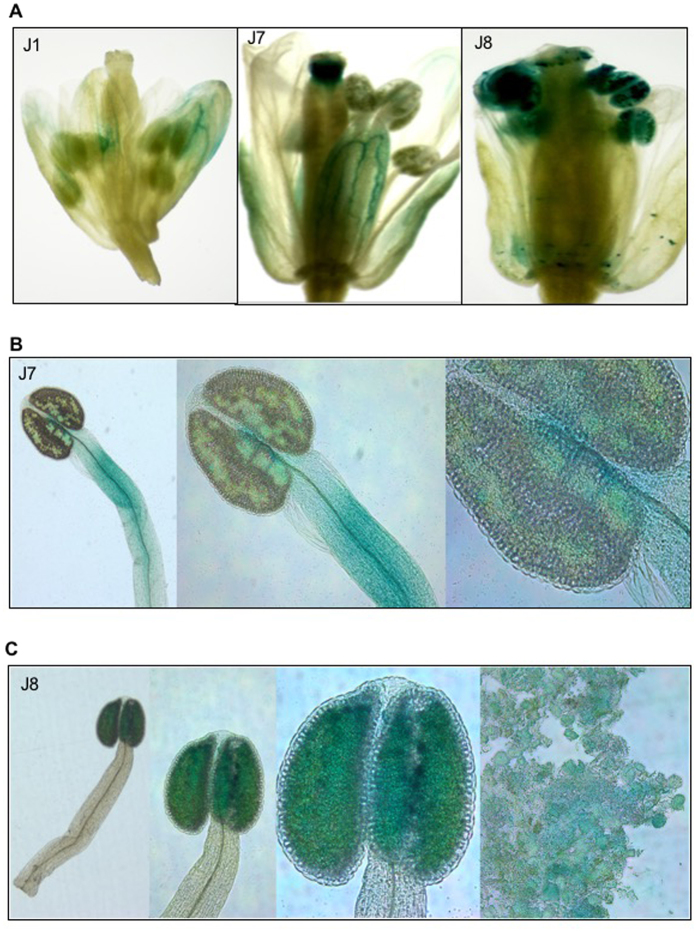
The promoter activity of *GmExo70J* genes in the floral organs. (**A**) Histochemical analysis of the promoter activities of *GmExo70J1* (J1), *GmExo70J7* (J7) and *GmExo70J8* (J8) in the floral organs of transgenic Arabidopsis flowers. (**B**) Histochemical analysis of the promoter activities of *GmExo70J7* (J7) in the filaments of stamen of transgenic Arabidopsis flowers. (**C**) Histochemical analysis of the promoter activity of *GmExo70J8* (J8) in the anthers and pollens of transgenic Arabidopsis flowers.

Promoter activities of *GmExo70J* genes in other reproductive tissues were also detected. For example, in transgenic Arabidopsis harboring the GUS gene driven by the *GmExo70J1* gene promoter, significant levels of the GUS activities were detected in the sepals of open flowers, particularly in the vascular tissues (Fig. 3A). Even stronger promoter activities of *GmExo70J7* were detected in the vascular tissues of sepals, filaments of stamen and stigma (Fig. 3A,B; Supplemental Fig. 1). On the other hand, very high promoter activities of the *GmExo70J8* gene were detected in anthers and pollens (Fig. 3A,C). The diverse expression patterns in different reproductive tissues suggest that GmExo70J proteins are involved in a range of important reproductive processes during floral organ and seed development.

### Functional analysis of *GmWRP1* using virus-induced gene silencing (VIGS)

**Figure 4:**
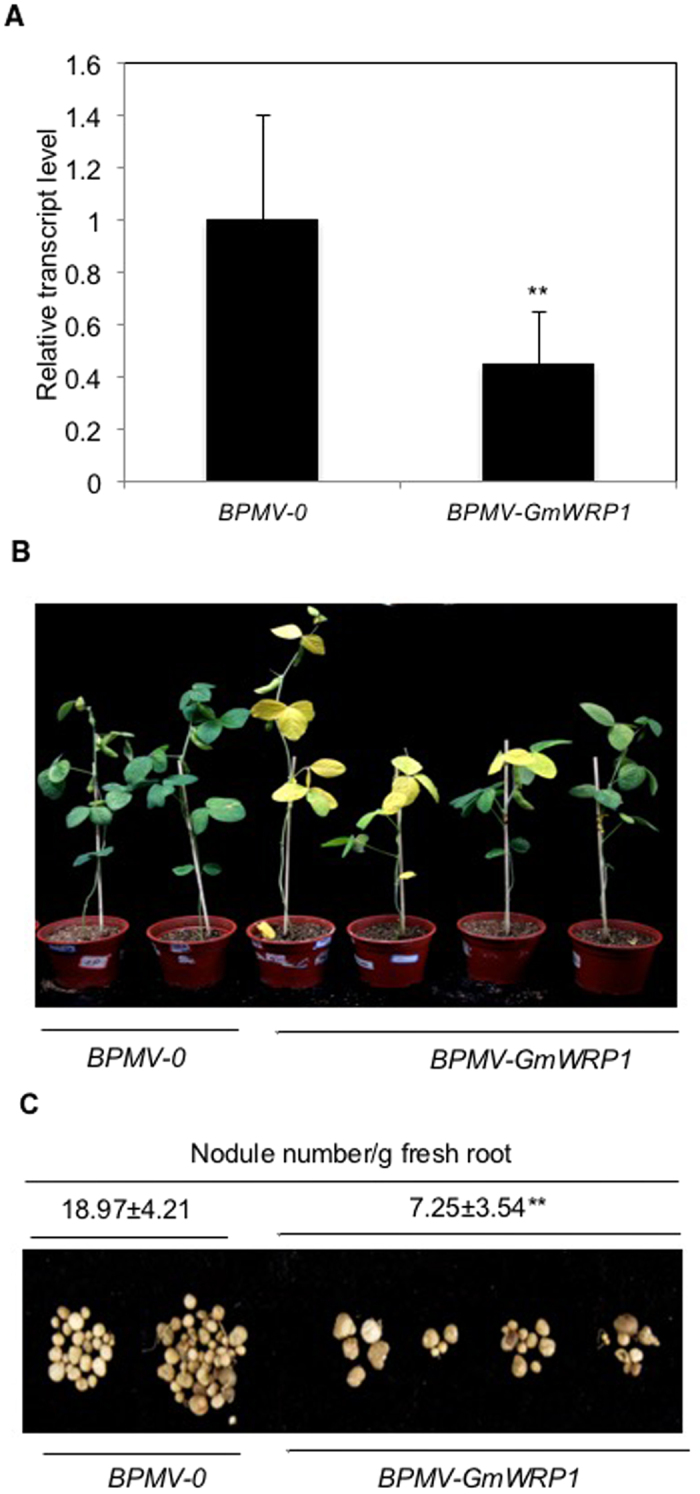
Phenotypes of *GmWRP1*-silenced plants. (**A)** Expression analysis of *GmWRP1* in silenced plants. Expression of *GmWRP1* was performed by qRT-PCR using a soybean actin gene as control. The results shown are from five individual empty BPMV vector control plants (BPMV-0) and *GmWRP1*-silenced plants (BPMV-GmWRP1). The asterisks indicate that the *GmWRP1* transcript levels in silenced plants are significantly different from those in control plants (*t* test, P = 0.01). (**B**) Early senescence of silenced plant leaves. The pictures of representative control plants inoculated with empty vectors (BPMV-0) and silenced plants inoculated with silencing vectors (BPMV-GmWRP1) were taken 7 weeks after BPMV inoculation. (**C**) Reduced nodule formation in silenced plants. The pictures of root nodules collected 7 weeks after BPMV inoculation from two representative control plants (BPMV-0) and four silenced plants (BPMV-GmWRP1) are shown. The number of nodules per gram of fresh root weight from at least five plants was also determined and shown above the picture. The asterisks indicate that the root nodule numbers in silenced plants are significantly different from those in control plants (*t* test, P = 0.01).

GmWRP1 contains a novel Golgi-targeted TM domain at its N-terminus and a WRKY domain at its C-terminal region that fails to bind W box sequences likely due to changes of a number amino acid residues at the conserved WRKY domain^7^. Close homologs of GmWRP1 are also found in other legumes but not in other plants. qRT-PCR analysis revealed expression of GmWRP1 in a wide range of soybean tissues include leaves, where its transcripts were substantially elevated with increased leaf age. In order to analyze its biological function directly, we attempted to silence *GmWRP1* in soybean using VIGS mediated by *Bean pod mottle virus* (BPMV), which has been successfully used for the functional investigation of a number of soybean genes^18^,^19^,^20^,^21^,^22^. A fragment of *GmWRP1* was cloned into the BPMV RNA2 silencing vector and introduced into soybean plants through bolistic bombardment together with the BPMV RNA1 vector. qRT-PCR analysis showed that the transcript level of GmWRP1 was substantially reduced in BPMV-WRP1-treated plants compared with control plants (Fig. 4A). Plants in which *GmWRP1* was silenced had displayed early senescence on the leaves, particularly those old leaves, when compared to control plants (Fig. 4B). Furthermore, the number of nodules per gram of fresh root weight was greatly reduced in *GmWRP1*-silenced soybean plants when compared to those in control plants (Fig. 4C). These observations suggest that GmWRP1 plays a positive role both in formation of root nodules and in delaying leaf senescence.

### Functional analysis of *GmExo70J* genes using VIGS

**Figure 5:**
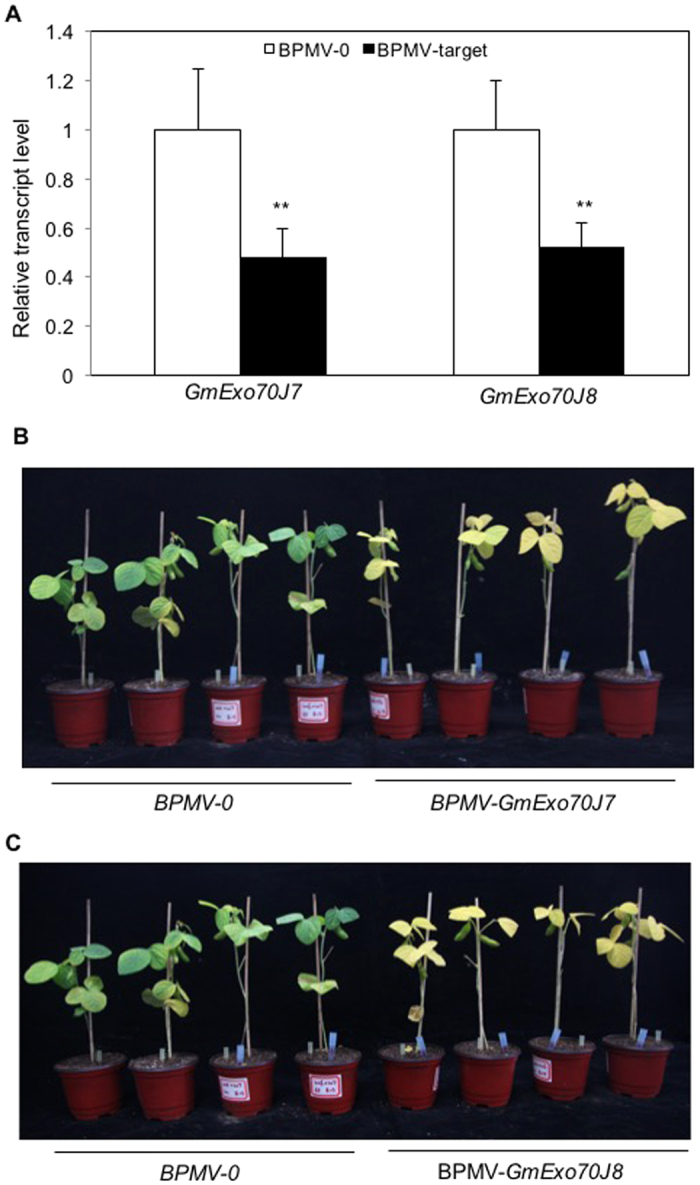
Premature leaf senescence in *GmExo70J7* and *GmExo70J8*-silenced plants. (**A**) Expression analysis of *GmExo70J7* and *GmExo70J8* in silenced plants. Expression of *GmExo70J7* and *GmExo70J8* was performed by qRT-PCR using a soybean actin gene as control. The results shown are from five individual plants inoculated with the empty BPMV vector (BPMV-0) or a BPMV derivative vector containing a target gene fragment for *GmExo70J7 GmExo70J8* (BPMV-target). The asterisks indicate that the *GmExo70J7* and *GmExo70J8* transcript levels in silenced plants are significantly different from those in control plants (*t* test, P = 0.01). (**B**) Early senescence of silenced plant leaves. The pictures of representative control plants inoculated with empty vectors (BPMV-0) and silenced plants inoculated with silencing vectors (*GmExo70J7* and *GmExo70J8*) were taken 7 weeks after BPMV inoculation.

We previously shown using qRT-PCR that transcript levels of soybean *GmExo70J* genes were also elevated in leaves with increased ages, suggesting their possible roles in leaf growth, maturation and/or senescence^7^. Using the promoter-GUS analysis, we have discovered that several *GmExo70J* genes including *GmExo70J7* and *GmExo70J8* are also expressed in reproductive organs (Figs 2 and 3). Because of their unique and interesting expression patterns not only in vegetative tissues but also in floral tissues, we also attempted to silence the two *GmExo70J* genes in soybean using VIGS to determine their biological functions. Fragments of the two *GmExo70J* genes were cloned into the BPMV RNA2 silencing vector and introduced into soybean plants through bolistic bombardment together with the BPMV RNA1 vector. qRT-PCR analysis showed that the transcript levels of *GmExo70J7* and *GmExo70J8* were substantially reduced in plants after bombardment with the respective silencing vectors when compared with control plants (Fig. 5A). Plants in which *GmExo70J7* or *GmExo70J8* was silenced displayed early senescence on the leaves (Fig. 5B,C), a phenotype similar to that of *GmWRP1*-silenced plants (Fig. 5B). This result suggests that not only GmWRP1 but also GmExo70J7 and GmExo70J8 play a critical role in the regulation of senescence of soybean leaves.

### Functional analysis of *GmExo70J* genes using artificial microRNA

Unlike *GmWRP*1, which is a single gene, *GmExo70J* genes belong to a subfamily of 12 members with a substantial degree of sequence identity^7^. As a result, off-target effects of virus-mediated silencing of a particular *GmExo70J* gene in soybean, particularly on those close sequence homologues, are likely. On the other hand, gene silencing through artificial microRNA (amiRNA) in transgenic plants has advantages over other RNAi-based gene silencing approaches, particularly in specificity^23^. While generation of stable transgenic soybean plants is difficult, transgenic soybean hairy roots can be readily obtained using *A. rhizogenes*-mediated procedures^24^ and therefore silencing of soybean genes in roots using amiRNA is very feasible. To determine the role of specific *GmExo70J* genes in symbiotic interactions in soybean roots, therefore, we tried to silence their expression using amiRNA in transgenic soybean hairy roots. Silencing of *GmExo70J* genes specifically in roots could also eliminate the indirect effects of silencing of the same genes in shoots, which occur with VIGS, so that their direct role in root symbiotic interactions can be assessed.

**Figure 6:**
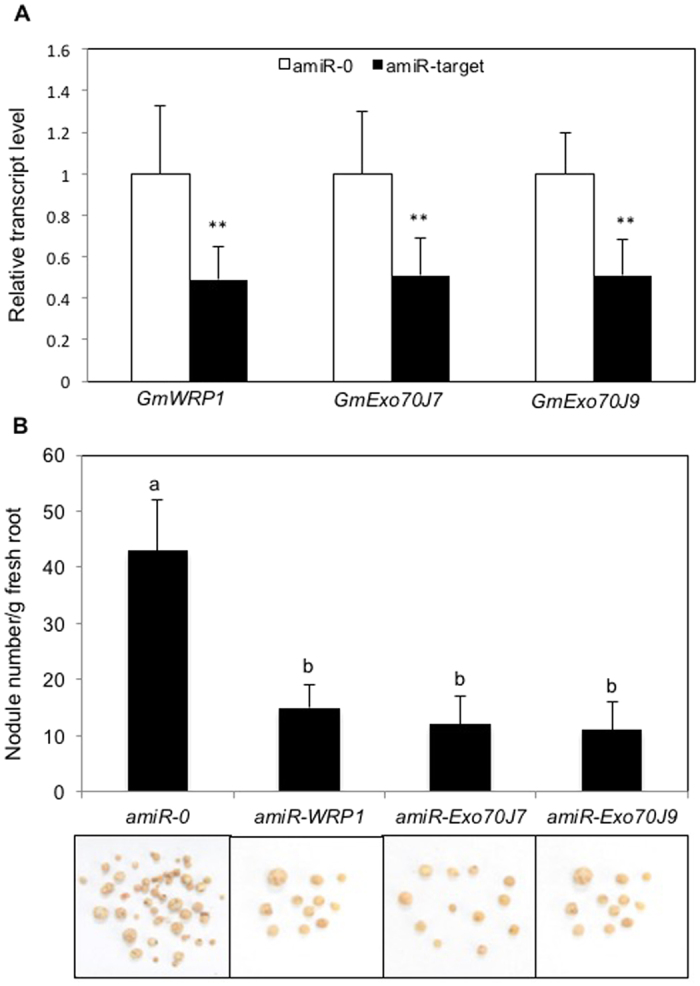
Reduced nodule formation in transgenic hairy roots expressing amiRNAs for *GmWRP1*, *GmExo70J7* and *GmExo70J9*. (**A**) Expression analysis of *GmWRP1* in silenced plants. Expression of *GmExo70J7* and *GmExo70J9* was performed by qRT-PCR using a soybean actin gene as control. The results shown are from five individual empty BPMV vector control plants (amiR-0) and *GmWRP1*, *GmExo70J7* or *GmExo70J9*-silenced plants (amiR-target). The asterisks indicate that the *GmWRP1*, *GmExo70J7* and *GmExo70J9* transcript levels in silenced plants are significantly different from those in control plants (*t* test, P = 0.01). (**B**) Reduced nodule formation in silenced plants. The fresh root weight and nodule number for each plant were determined from representative control plants (amiR-0) and silenced plants each for *GmWRP1 (amiR-WRP1*), *GmExo70J7 (amiR-Exo70J7*) and *GmExo70J9 (amiR-Exo70J9*) to calculate the number of nodules per gram of fresh root weight. According to Duncan’s multiple range test (P = 0.01), means do not differ significantly if they are indicated with the same letter. The pictures of root nodules collected 5 weeks after inoculation from a representative plant for each gene are also shown.

Among the 12 *GmExo70J* genes identified, seven encode Exo70 proteins with a novel TM domain targeted to Golgi apparatus^7^. To determine their roles in symbiotic interactions in soybean roots, we generated gene-specific amiRNA constructs for these seven TM-containing *GmExo70J* genes in pSM103, an intron-GFP7 Agrobacterium-compatible binary vector for amiRNA-mediated gene silencing in legume roots^25^. A gene-specific amiRNA construct was also generated for *GmWRP1* for comparison with VIGS. The cotyledonary nodes of soybean seedlings were infected by *A. rhizogenes* K599 containing the pSM103 derivatives carrying one of eight different amiRNAs. GFP-positive transgenic roots were readily identified (Supplemental Fig. 2) and qRT-PCR confirmed reduced transcript levels of the targeted genes in the transgenic hairy roots (Fig. 6A). The composite soybeans with GFP-positive hairy roots were inoculated with rhizobia and the numbers of nodules was determined 35 days post inoculation. As shown in Fig. 6B, the number of nodules per gram fresh root in the transgenic hairy roots containing *amiRNA-GmWRP1* was reduced by 3-fold when compared to that of control hairy roots infected with an empty amiRNAi vector. This level of reduction in nodulation by amiRNA-mediated silencing of *GmWRP1* was very similar to that in the roots following VIGS of the same gene (Fig. 4C). Likewise, we observed 3- to 4-fold reduction of nodulation in the transgenic hairy roots expressing amiRNAs that silence *GmExo70J7* or *GmExo70J9* (Fig. 6). By contrast, no significant reduction in nodule numbers were observed in the transgenic hairy roots containing amiRNAs for the other five genes encoding GmExo70J proteins containing the novel N-terminal domain (data not shown). Thus, GmWRP1 and some of the GmExo70J subfamily members play a critical role in the nodulation of soybean roots.

### Expression of *GmExo70J*7 and *GmExo70J9* in response to rhizobia

**Figure 7:**
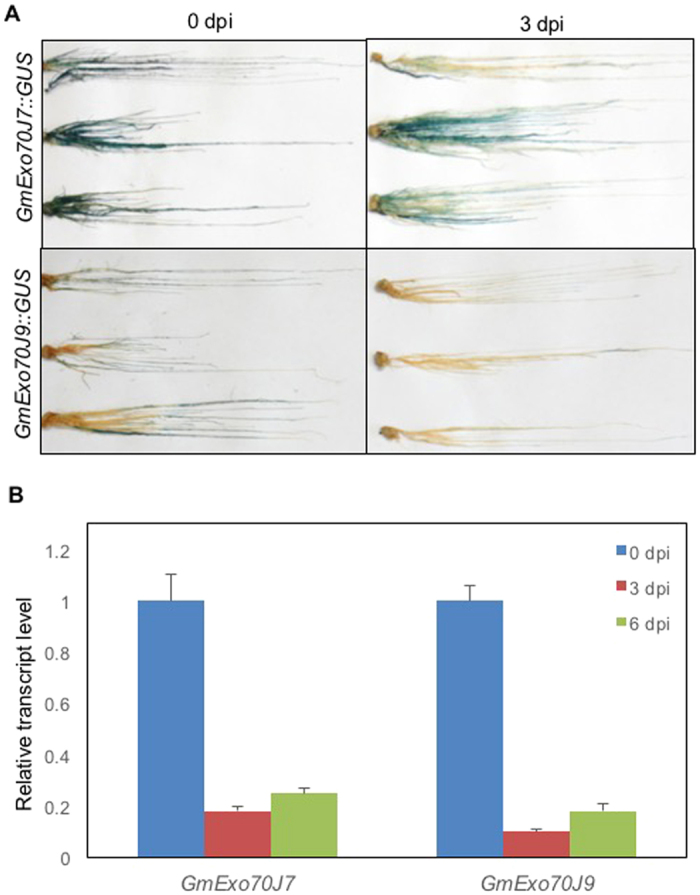
The promoter activity of *GmExo70J* genes in response to rhizobial infection. (**A**) Histochemical analysis of the promoter activities of *GmExo70J7 (GmExo70J7::GUS*) and *GmExo70J9 (GmExo70J9::GUS*) in transgenic soybean hairy roots at 0 and 3 days post inoculation of rhizobia (dpi). (**B**) qRT-PCR analysis of *GmExo70J7* and *GmExo70J9* in soybean roots at 0, 3 and 6 dpi of rhizobia.

Reduced nodule formation in *GmExo70J7*- and *GmExo70J9*-silenced hairy roots suggests that the two genes have important roles in the symbiotic interactions of soybean roots with rhizobia. To further determine their involvement in the symbiotic interaction of soybean roots, we analyzed their expression in response to rhizobia infection. The promoter-GUS constructs for the two genes were transformed into soybean hairy roots using the *A. rhizogenes* K599 strain. The obtained hairy roots were then inoculated with rhizobia and the GUS activities were assayed at 0 and 3 days post inoculation (dpi). As shown in Fig. 7A, strong promoter activity of *GmExo70J7* was detected in the soybean hairy roots, as observed in roots of transgenic Arabidopsis plants harboring the same construct (Fig. 1). In rhizobia-inoculated roots, the promoter activity of the gene was again readily detected but the activity was significant reduced when compared with that in mock inoculated roots based on the intensities of GUS staining (Fig. 7A). In the transgenic soybean hairy roots containing the promoter-GUS construct for *GmExo70J9*, only low levels of GUS activity were detected (Fig. 7A), again consistent with the data in transgenic Arabidopsis roots containing the same construct (Fig. 1). At 3 dpi, the GUS activity in the roots containing the construct driven by the *GmExo70J9* promoter was even lower than at 0 dpi (Fig. 7A). Thus, the promoter activities for both *GmExo70J7* and *GmExo70J9* appeared to be down regulated upon infection of rhizobia. Analysis of the transcript levels for the two genes using qRT-PCR showed that at 3 and 6 dpi, the transcript levels for *GmExo70J7* and *GmExo70J9* were only about 20% of those at 0 dpi, confirming their down-regulation in soybean roots upon rhizobial infection (Fig. 7B).

## Discussion

We have recently reported identification of a novel WRKY-related protein (GmWRP1) and a new subfamily of Exo70 proteins (GmExo70J) from soybean with homologs found only in legumes^7^. Given their legume-specific nature, these novel WRKY and Exo70 protein variants could have evolved for the specific process of symbiotic interactions between legumes and rhizobia. In fact, it has been recently reported that an Exo70I protein from *Medicago truncatula* with homologs present exclusively in plant forming arbuscular mycorrhizal symbiosis is required for development of a sub-domain of the periarbuscular membrane during arbuscular mycorrhizal symbiosis^26^. To address the roles of the legume-specific *GmWRP1* and *GmExo70J* genes, we have sought a better knowledge about their expression patterns. We have also tried to determine directly their roles in soybean by suppressing their expression using virus- and artificial microRNA-mediated gene silencing. The data from these experiments suggest a broad role of these WRKY and Exo70 protein variants not only in root nodulation but also in the processes of plant growth and development.

Plants are unique in term of the multiplication of *Exo70* genes, which may allow for formation of different exocyst complexes with different functions. The diverse expression patterns of Arabidopsis *AtExo70* genes and the distinct phenotypes of Arabidopsis mutants for a number of its *AtExo70* genes support the functional divergence and specificity of plant Exo70 protein family members^14^,^15^,^27^,^28^,^29^,^30^,^31^. Interestingly, expression of Arabidopsis *AtExp70* gene members is tightly associated with cells actively undergoing elongation or differentiation including root hairs and pollen tubes^16^. In the mature organs and tissues, *AtExo70* expression was hardly detectable^16^. Like Arabidopsis *AtExo70* genes each expressing specifically in one or several cell types^16^, the 12 soybean *GmExo70J* genes also have highly diverse expression levels and tissue-specific expression patterns (Figs 1, 2, 3), suggesting extensive functional divergence even within the same clade of the gene family. In addition, as with Arabidopsis *AtExo70* genes, we observed expression of some *GmExo70* genes in cells undergoing active cell elongation and differentiation such as filaments of stamen, stigma, anthers and pollens (Fig. 3). Thus at least some of the legume-specific Exo70J proteins appear to be involved in exocytosis during active cell growth and differentiation. In contrast to the general absence of expression of Arabidopsis *AtExo70* genes in mature organs and tissues^16^, however, we observed expression of *GmExo70J* genes in mature leaves, roots and floral organs (Figs 1, 2, 3). In fact, qRT-PCR analysis indicated that expression of a number of *GmExo70J* genes and *GmWRP1* was elevated greatly in soybean leaves with increased leaf age^7^. In addition, the promoter activities of *GmExo70J1*, *GmExo70J4, GmExo70J6, GmExo70J7* and *GmExo70J8* increased substantially in the floral organ-supporting receptacles during the development and maturation of siliques (Fig. 2). The promoter activities for *GmExo70J1*, *GmExo70J6* and *GmExo70J7* were also particularly strong in the vascular tissues of shoots, filaments of stamen and sepals (Figs 2 and 3; Supplemental Fig. 1). The strongly preferential expression in the vascular system of mature organs and tissues raise the possibility of a major role of these legume-specific Exo70J proteins in transport of nutrients and other types of biological molecules. Thus, the expression of *GmExo70J* genes is not restricted to roots or to those elongating and differentiating cells. The enhanced expression of some of the GmExo70J genes in aged leaves, reproductive organs and vascular tissues suggest their broad roles in plant growth and development.

Direct functional analysis through gene silencing revealed that *GmWRP1* and some of the *GmExo70J* genes function as negative regulators of soybean leaf senescence (Figs 4 and 5). The premature senescence of these *GmExo70J*-silenced soybean plants is similar to the mutants defective in autophagy, which is induced in plant leaves with increased age^32^. In soybean, leaf senescence at reproductive stage is also associated with induction of autophagy-related genes, *GmATG8c*, *GmATG8i* and *GmATG4*^33^. Plant senescence is a developmental process that serves, among other functions, as a process for nutrient redistribution^34^. In the older parts of plants such as lower leaves, autophagy could participate in the degradation of defective and unneeded cellular structures and molecules including chloroplasts and chloroplast proteins for efficient nutrient relocalization and utilization by young tissues and developing fruits and seeds^32^,^35^,^36^. In autophagy-deficient mutants, lack of degradation of the defective and unneeded cellular structures and molecules presumably can lead to cellular stress, accumulation of reactive oxygen species and salicylic acid, which could operate through a positive feedback loop to trigger early senescence and programmed cell death^32^. Likewise, we observed increased expression of the *GmWRP1* and *GmExo70J* genes with increased leaf ages^7^. Silencing of *GmWRP1* and *GmExo70J* genes could lead to early senescence of soybean leaves through a similar process involving defects in modification and transport of proteins that can potentially lead to proteotoxic stress and cell death. In addition, GmWRP1 and GmExo70J proteins could be involved directly or indirectly in the transport of regulatory molecules including plant hormones. For example, GmExo70J proteins could regulate the level and distribution of auxin through their roles in the trafficking of auxin transporters between the plasma membrane and the endosomes. The exocyst complex is involved in PIN auxin efflux carrier recycling and polar auxin transport in Arabidopsis^37^.

Since leaf senescence is a common process in all plants, it is intriguing that regulation of this important program in soybean, and perhaps in other legumes as well, requires a unique group of proteins not present in other plants. This observation suggests that the leaf senescence program in legumes may differ significantly from those of other plants, likely due to differences in nitrogen assimilation, transport and redistribution. Nitrogen in seeds is accumulated during the grain-filling stage largely through remobilization from vegetative organs of leaves and stems^38^. At the onset of grain filling of other plants, there is an increased demand for nitrogen in seeds but a decrease in uptake of nitrogen by roots as plants progress to maturity with reduced transport of carbohydrates to the roots^39^. Decreased nitrogen uptake during the grain-filling period would make it necessary to enhance nutrient remobilization from leaves and the stem for grain-filling, eventually leading to leaf senescence. Because of the symbiotic nitrogen fixation by their roots, there will be less demand for remobilized nitrogen from leaves of legumes, particularly during early reproductive stages. In fact, maintaining high nitrogen levels and delaying leaf senescence could sustain high rates in both photosynthesis in leaves and transport of carbohydrates to the roots for high rates of nitrogen fixation. Legume-specific GmWRP1 and GmExo70J proteins could be part of the important genetic program that modulates the remobilization of nutrients and onset of senescence in leaves through regulation of endosome trafficking of important biological molecules including, for example, proteins and hormones.

Silencing of *GmWRP1*, *GmExo70J7* and *GmExo70J9* led to reduction in nodule numbers in soybean roots (Figs 4 and 6). This important finding supports that the biological functions of the legume-specific proteins are directly associated with the cellular processes uniquely important to legume plants: their association with symbiotic rhizobia for nitrogen fixation. Interestingly, promoter-GUS analysis revealed that the expression of *GmExo70J* and *GmExo70J9* was actually down regulated in transgenic soybean hairy roots after rhizobia infection (Fig. 7). One possible explanation for this seemingly contradictory result is the differential requirement for the abundance of these exocyst subunits at different stages of the symbiotic relationship. For example, relatively high levels of the two Exo70J proteins may be required during the very early stages of nodulation but not during the late stages of nodule formation. Alternatively, down-regulation of positive regulators of nodule formation during nodulation could be a form of feedback control. Rhizobum infection could lead to the formation of innumerable nodules, which would not be supported metabolically by the host plant. A systemic control of nodule development possibly through a feedback mechanism has long been proposed^40^,^41^.

Nodulation starts with the induction of rhizobial *nod* genes in response to specific flavonoids secreted by the roots of legume plants^42^,^43^. Expression of the *nod* genes results in production of Nod factors by rhizobial bacteria, which induce deformation and curling of root hairs. Following local hydrolysis of plant cell walls in the curled region, the plasma membrane invaginates and new plant cell wall materials are deposited, resulting in the formation of the infection thread, by which the bacteria enter the plant roots. Concomitant with infection thread formation, inner cortical cells are mitotically reactivated, forming the nodule primordium. Infection threads grow toward this primordium and once there, release bacteria into the cytoplasm. Within the infected cells, the rhizobia divide and enlarge to form bacteroids, which are separated from the plant cytoplasm by the peribacteroid membrane. GmExo70J-depednent exocytosis and more broad endosome trafficking can participate in some of these important processes during the early stages of nodulation that require secretion of biological molecules (e.g. flavonoids), membrane reorganization, cell division and differentiation. Altered leaf growth and morphology of transgenic Arabidopsis plants expressing some of the soybean *GmExo70J* genes support a possible role of these proteins in regulation cell division and expansion. Furthermore, both Arabidopsis AtExo70B proteins (AtExo70B1 and AtExo70B2) play roles in plant immune responses to microbial pathogens^30^,^31^,^44^. Arabidopsis Exo70B1 is also required for autophagy-related membrane traffic to the vacuole^30^, a process implicated in a range of plant processes including plant-microbe interactions^45^. As legume-specific Exo70J proteins are evolutionarily closely related to Exo70B proteins^7^, some legume Exo70J proteins could be directly involved in the communication between legumes and rhizobial bacteria.

Symbiotic nitrogen fixation takes place in bacteroids in infected legume nodule cells, catalyzed by the bacterial enzyme nitrogenase. Highly active transport and metabolism occurs in an infected nodule cell^46^. To fuel nitrogen fixation, sucrose from the shoot is converted to malate in the plant and imported across the symbiosome membrane and into bacteroids. Ammonia, the product of the nitrogen fixation, is exported back to the plant, where it is assimilated into nitrogen compounds for export to the shoot. The plant also must provide other nutrients including metals and ions to the bacteroid. The rapid exchange of organic compounds between the host plants and the rihzobial partners relies on the transport systems operative within the individual nodule. In a variety of plant tissues, *GmExo70J* genes are expressed predominantly in the vascular tissues (Figs 1 and 3; Supplemental Fig. 1), suggesting a critical role in transport of biological molecules. Some of the *GmEx070J* genes including *GmExo70J7* are also expressed at relatively high levels in roots (Fig. 1). Therefore, GmExo70J-mediated vesicle trafficking could also be involved in the uptake, secretion and transport of nitrogen, nitrogen-containing compounds and photosynthates within the individual nodules. Further studies of the unique Exo70 proteins through analysis of their associated exocyst complex subunits, tissue-specific expression patterns, dynamic subcellular localization and, most importantly, isolation and characterization of mutants through, for example, gene editing technology, will be necessary to fully understand the unique roles of the subfamily of Exo70J proteins in legume plants.

## Methods

### Plant materials and growth conditions

Soybean (*Glycine max* cv. ‘Williams 82’) and Arabidopsis (Col-0) plants were grown in a greenhouse or growth room at 25 °C with a photoperiod of 12 h.

### Generation of *GmExo70J::GUS* and silencing constructs

For generation of *GmExo70J::GUS* constructs, the ~1.5 kb genomic sequences upstream of the start codons of the 12 *GmExo70J* genes were amplified by PCR using the primers listed in Supplemental Table 1. The amplified PCR fragments were cloned upstream of the β-glucuronidase (GUS) reporter gene in a binary vector. For generating constructs for virus-induced gene silencing (VIGS), DNA fragments of *GmWRP1* and *GmExo70J* genes were PCR-amplified using gene-specific primers listed in Supplemental Table 2 and cloned into the BPMV RNA2 silencing vector^20^,^47^. Artificial microRNA vectors for GmWRP1 and GmExo70J genes were constructed in PSM103^25^ with overlapping PCR using gene-specific primers designed using WMD3 (http://wmd3.weigelworld.org/cgi-bin/webapp.cgi?page=Home;project=stdwmd) (Supplemental Table 2). Correct sequences and fusion of the constructs were confirmed by DNA sequencing.

### Arabidopsis transformation

For generating transgenic Arabidopsis plants, *GmExo70J::GUS* plasmids were transformed into Col-0 wild-type plants using *Agrobacterium*-mediated floral-dip procedure^48^. Transformants were identified for resistance to Basta.

### GUS histochemical staining

The GUS histochemical staining of Arabidopsis plants and soybean transgenic hairy roots was performed as previously described^49^.

### BPMV-mediated gene silencing

Inoculation of soybean seedlings at V1 stage with DNA-based BPMV constructs via biolistic particle bombardments was performed as described previously^20^,^47^. At 3 weeks post-virus inoculation, the third trifoliolate were harvested to isolate RNA for detection of BPMV by reverse transcription-PCR and verification of gene silencing by qRT-PCR.

### Generation of transgenic soybean hairy roots

Transgenic soybean hairy roots were generated as described previously^24^. The cotyledonary nodes of 3–5 days old soybean seedlings were infected by *Agrobacterium rhizogenes* K599 containing recombinant vectors. After cultivating at humidity condition for about two weeks, composite soybean plants with transgenic hair roots were transferred to soil.

### Analysis of nodulation in transgenic soybean hairy roots

The rhizobacteria used in the experiments were isolated from the nodules of soybean roots grown in the greenhouse at Zhejiang University. The nodules were cut from soybean roots and sterilized in 20% Clorox for 5 minutes. After washed with water, the sterile nodules were put into LB with 0.3 M glucose and then crushed by sterile pestle. The bacteria were maintained at 28 °C on the selective YMA (mannitol 3 g/L, MgSO_4_·7H_2_O 0.2 g/L, NaCl 0.1 g/L, K_2_HPO_4_ 0.25 g/L, KH_2_PO_4_ 0.25 g/L, agar 15 g/L, pH 6.8) medium with 0.003% Congo red dye. The 16S rRNA fragment of the bacteria was PCR-amplified using universal 16S rRNA-specific primers (5′-AGAGTTTGATCCTGGCTCAG-3′ and 5′-GGTTACCTTGTTACGACTT-3′) and sequenced. The bacterium was identified as *Sinorhizobium* based on the 16S rRNA sequence. For inoculation, a single bacterial colony was grown in YMA liquid medium at 28 °C. The bacterial suspension with OD_600_ = 1.0 in H_2_O was used for inoculation (10 ml/plant).

### qRT-PCR

Arabidopsis and soybean samples were lyophilized and stored at −80 °C until use. Total RNA was isolated from plant tissues using the Trizol reagent according to the supplier’s instruction. Extracted RNA was treated with DNase to remove contaminating DNA and reverse transcribed using the ReverTran Ace^®^ qPCR RT kit (Toyobo) for reverse transcriptase-PCR. qRT-PCR was performed with an StepOnePlus™ Real-Time PCR System (ABI). PCRs were performed using the SYBR^®^ Green qPCR Master Mixes (Takara) and gene-specific primers listed in Supplemental Table 3. Relative gene expression was calculated as previously described^50^. Soybean *Actin* gene (Glyma18 g52780, 5′-GTGCACAATTGATGGACCAG-3′ and 5′-GCACCACCGGAGAGAAAATA-3′) was used as internal control.

## Additional Information

**How to cite this article**: Wang, Z. *et al.* Expression and Functional Analysis of a Novel Group of Legume-specific WRKY and Exo70 Protein Variants from Soybean. *Sci. Rep.* **6**, 32090; doi: 10.1038/srep32090 (2016).

## Supplementary Material

Supplementary Information

## References

[b1] Martinez-Romero E. & Caballero-Mellado J. Rhizobium phylogenies and bacterial genetic diversity. Crit. Revs. Plant Sci. 15, 113–140 (1996).

[b2] Graham P. H. & Vance C. P. Legumes: importance and constraints to greater use. Plant physiology 131, 872–877, doi: 10.1104/pp.017004 (2003). 12644639 PMC1540286

[b3] O’Rourke J. A., Bolon Y. T., Bucciarelli B. & Vance C. P. Legume genomics: understanding biology through DNA and RNA sequencing. Annals of Botany 113, 1107–1120, doi: 10.1093/aob/mcu072 (2014). 24769535 PMC4030821

[b4] Schmutz J. *et al.* Genome sequence of the palaeopolyploid soybean. Nature 463, 178–183, doi: 10.1038/nature08670 (2010). 20075913

[b5] Libault M. *et al.* Legume transcription factor genes: what makes legumes so special? Plant physiology 151, 991–1001, doi: 10.1104/pp.109.144105 (2009). 19726573 PMC2773095

[b6] Graham M. A., Silverstein K. A., Cannon S. B. & VandenBosch K. A. Computational identification and characterization of novel genes from legumes. Plant physiology 135, 1179–1197, doi: 10.1104/pp.104.037531 (2004). 15266052 PMC519039

[b7] Chi Y. *et al.* Identification and characterization of a novel group of legume-specific, Golgi apparatus-localized WRKY and Exo70 proteins from soybean. Journal of experimental botany 66, 3055–3070, doi: 10.1093/jxb/erv104 (2015). 25805717 PMC4449531

[b8] Samuel M. A. *et al.* Cellular pathways regulating responses to compatible and self-incompatible pollen in Brassica and Arabidopsis stigmas intersect at Exo70A1, a putative component of the exocyst complex. The Plant cell 21, 2655–2671, doi: 10.1105/tpc.109.069740 (2009). 19789280 PMC2768929

[b9] Boyd C., Hughes T., Pypaert M. & Novick P. Vesicles carry most exocyst subunits to exocytic sites marked by the remaining two subunits, Sec3p and Exo70p. The Journal of cell biology 167, 889–901, doi: 10.1083/jcb.200408124 (2004). 15583031 PMC2172445

[b10] Guo W., Grant A. & Novick P. Exo84p is an exocyst protein essential for secretion. The Journal of biological chemistry 274, 23558–23564 (1999). 10438536 10.1074/jbc.274.33.23558

[b11] Guo W., Roth D., Walch-Solimena C. & Novick P. The exocyst is an effector for Sec4p, targeting secretory vesicles to sites of exocytosis. The EMBO journal 18, 1071–1080, doi: 10.1093/emboj/18.4.1071 (1999). 10022848 PMC1171198

[b12] Finger F. P., Hughes T. E. & Novick P. Sec3p is a spatial landmark for polarized secretion in budding yeast. Cell 92, 559–571 (1998). 9491896 10.1016/s0092-8674(00)80948-4

[b13] Chong Y. T. *et al.* Characterization of the Arabidopsis thaliana exocyst complex gene families by phylogenetic, expression profiling, and subcellular localization studies. The New phytologist 185, 401–419, doi: 10.1111/j.1469-8137.2009.03070.x (2010). 19895414

[b14] Synek L. *et al.* AtEXO70A1, a member of a family of putative exocyst subunits specifically expanded in land plants, is important for polar growth and plant development. The Plant journal: for cell and molecular biology 48, 54–72, doi: 10.1111/j.1365-313X.2006.02854.x (2006). 16942608 PMC2865999

[b15] Li S. *et al.* EXO70A1-mediated vesicle trafficking is critical for tracheary element development in Arabidopsis. The Plant cell 25, 1774–1786, doi: 10.1105/tpc.113.112144 (2013). 23709627 PMC3694705

[b16] Li S. *et al.* Expression and functional analyses of EXO70 genes in Arabidopsis implicate their roles in regulating cell type-specific exocytosis. Plant physiology 154, 1819–1830, doi: 10.1104/pp.110.164178 (2010). 20943851 PMC2996038

[b17] Smyth D. R., Bowman J. L. & Meyerowitz E. M. Early flower development in Arabidopsis. The Plant cell 2, 755–767, doi: 10.1105/tpc.2.8.755 (1990). 2152125 PMC159928

[b18] Liu J. Z. *et al.* Positive and negative roles for soybean MPK6 in regulating defense responses. Molecular plant-microbe interactions: MPMI 27, 824–834, doi: 10.1094/MPMI-11-13-0350-R (2014). 24762222

[b19] Kandoth P. K. *et al.* A virus-induced gene silencing method to study soybean cyst nematode parasitism in Glycine max. BMC research notes 6, 255, doi: 10.1186/1756-0500-6-255 (2013). 23830484 PMC3708766

[b20] Zhang C., Bradshaw J. D., Whitham S. A. & Hill J. H. The development of an efficient multipurpose bean pod mottle virus viral vector set for foreign gene expression and RNA silencing. Plant physiology 153, 52–65, doi: 10.1104/pp.109.151639 (2010). 20200069 PMC2862437

[b21] Zhang C., Yang C., Whitham S. A. & Hill J. H. Development and use of an efficient DNA-based viral gene silencing vector for soybean. Molecular plant-microbe interactions: MPMI 22, 123–131, doi: 10.1094/MPMI-22-2-0123 (2009). 19132865

[b22] Liu J. Z. *et al.* Soybean homologs of MPK4 negatively regulate defense responses and positively regulate growth and development. Plant physiology 157, 1363–1378, doi: 10.1104/pp.111.185686 (2011). 21878550 PMC3252160

[b23] Schwab R., Ossowski S., Riester M., Warthmann N. & Weigel D. Highly specific gene silencing by artificial microRNAs in Arabidopsis. The Plant cell 18, 1121–1133, doi: 10.1105/tpc.105.039834 (2006). 16531494 PMC1456875

[b24] Kereszt A. *et al.* Agrobacterium rhizogenes-mediated transformation of soybean to study root biology. Nat Protoc 2, 948–952, doi: 10.1038/nprot.2007.141 (2007). 17446894

[b25] Melito S. *et al.* A nematode demographics assay in transgenic roots reveals no significant impacts of the Rhg1 locus LRR-Kinase on soybean cyst nematode resistance. BMC plant biology 10, 104, doi: 10.1186/1471-2229-10-104 (2010). 20529370 PMC3095272

[b26] Zhang X., Pumplin N., Ivanov S. & Harrison M. J. EXO70I Is Required for Development of a Sub-domain of the Periarbuscular Membrane during Arbuscular Mycorrhizal Symbiosis. Curr Biol 25, 2189–2195, doi: 10.1016/j.cub.2015.06.075 (2015). 26234213

[b27] Zhang C. *et al.* Endosidin2 targets conserved exocyst complex subunit EXO70 to inhibit exocytosis. Proceedings of the National Academy of Sciences of the United States of America 113, E41–E50, doi: 10.1073/pnas.1521248112 (2016). 26607451 PMC4711834

[b28] Hong D., Jeon B. W., Kim S. Y., Hwang J. U. & Lee Y. The ROP2-RIC7 pathway negatively regulates light-induced stomatal opening by inhibiting exocyst subunit Exo70B1 in Arabidopsis. The New phytologist 209, 624–635, doi: 10.1111/nph.13625 (2016). 26451971

[b29] Ding Y. *et al.* Exo70E2 is essential for exocyst subunit recruitment and EXPO formation in both plants and animals. Molecular biology of the cell 25, 412–426, doi: 10.1091/mbc.E13-10-0586 (2014). 24307681 PMC3907280

[b30] Kulich I. *et al.* Arabidopsis exocyst subcomplex containing subunit EXO70B1 is involved in autophagy-related transport to the vacuole. Traffic 14, 1155–1165, doi: 10.1111/tra.12101 (2013). 23944713

[b31] Pecenkova T. *et al.* The role for the exocyst complex subunits Exo70B2 and Exo70H1 in the plant-pathogen interaction. Journal of experimental botany 62, 2107–2116, doi: 10.1093/jxb/erq402 (2011). 21199889 PMC3060688

[b32] Yoshimoto K. *et al.* Autophagy negatively regulates cell death by controlling NPR1-dependent salicylic acid signaling during senescence and the innate immune response in Arabidopsis. Plant Cell 21, 2914–2927, doi: tpc.109.068635 [pii] 10.1105/tpc.109.068635 (2009). 19773385 PMC2768913

[b33] Htwe N. M. P. S. *et al.* Leaf senescence of soybean at reproductive stage is associated with induction of autophagy-related genes, GmATG8c, GmATG8i and GmATG4. Plant Production Science 14, 141–147 (2015).

[b34] Woo H. R., Kim H. J., Nam H. G. & Lim P. O. Plant leaf senescence and death - regulation by multiple layers of control and implications for aging in general. Journal of cell science 126, 4823–4833, doi: 10.1242/jcs.109116 (2013). 24144694

[b35] Bassham D. C. Plant autophagy–more than a starvation response. Current opinion in plant biology 10, 587–593, doi: 10.1016/j.pbi.2007.06.006 (2007). 17702643

[b36] Hanaoka H. *et al.* Leaf senescence and starvation-induced chlorosis are accelerated by the disruption of an Arabidopsis autophagy gene. Plant Physiol 129, 1181–1193, doi: 10.1104/pp.011024 (2002). 12114572 PMC166512

[b37] Drdova E. J. *et al.* The exocyst complex contributes to PIN auxin efflux carrier recycling and polar auxin transport in Arabidopsis. Plant J 73, 709–719, doi: 10.1111/tpj.12074 (2013). 23163883

[b38] Pan W. L., Camberato J. J., Jackson W. A. & Moll R. H. Utilization of Previously Accumulated and Concurrently Absorbed Nitrogen during Reproductive Growth in Maize: Influence of Prolificacy and Nitrogen Source. Plant physiology 82, 247–253 (1986). 16665001 10.1104/pp.82.1.247 PMC1056098

[b39] Christensen L. E., Below F. E. & Hageman R. H. The effects of ear removal on senescence and metabolism of maize. Plant physiology 68, 1180–1185 (1981). 16662071 10.1104/pp.68.5.1180 PMC426065

[b40] Loh J. T. & Stacey G. Feedback regulation of the Bradyrhizobium japonicum nodulation genes. Molecular microbiology 41, 1357–1364 (2001). 11580840 10.1046/j.1365-2958.2001.02603.x

[b41] Caetano-Anolles G., Joshi P. A. & Gresshoff P. M. Spontaneous nodules induce feedback suppression of nodulation in alfalfa. Planta 183, 77–82, doi: 10.1007/BF00197570 (1991). 24193536

[b42] Long S. R. Symbiosis: Receptive to infection. Nature 523, 298–299, doi: 10.1038/nature14632 (2015). 26153862

[b43] Long S. R. Genes and signals in the rhizobium-legume symbiosis. Plant physiology 125, 69–72 (2001). 11154299 10.1104/pp.125.1.69 PMC1539328

[b44] Stegmann M. *et al.* The exocyst subunit Exo70B1 is involved in the immune response of Arabidopsis thaliana to different pathogens and cell death. Plant Signaling & Behavior 8, e27421, doi: 10.4161/psb.27421 (2013). 24389869 PMC4091220

[b45] Zhou J., Yu J. Q. & Chen Z. The perplexing role of autophagy in plant innate immune responses. Mol Plant Pathol 15, 637–645, doi: 10.1111/mpp.12118 (2014). 24405524 PMC6638830

[b46] Udvardi M. & Poole P. S. Transport and metabolism in legume-rhizobia symbioses. Annual review of plant biology 64, 781–805, doi: 10.1146/annurev-arplant-050312-120235 (2013). 23451778

[b47] Zhang C., Whitham S. A. & Hill J. H. Virus-induced gene silencing in soybean and common bean. Methods in molecular biology 975, 149–156, doi: 10.1007/978-1-62703-278-0_11 (2013). 23386301

[b48] Clough S. J. & Bent A. F. Floral dip: a simplified method for Agrobacterium-mediated transformation of Arabidopsis thaliana. Plant J 16, 735–743 (1998). 10069079 10.1046/j.1365-313x.1998.00343.x

[b49] Crone D., Rueda J., Martin K. L., Hamilton D. A. & Mascarenhas J. P. The differential expression of a heat shock promoter in floral and reproductive tissues. Plant, Cell and Environment. 24, 869–874 (2001).

[b50] Livak K. J. & Schmittgen T. D. Analysis of relative gene expression data using real-time quantitative PCR and the 2(-Delta Delta C(T)) Method. Methods 25, 402–408, doi: 10.1006/meth.2001.1262 (2001). 11846609

