# Supplementary material for: Expression and Functional Analysis of a Novel Group of Legume-specific WRKY and Exo70 Protein Variants from Soybean
Source: Sci Rep. 2016 Aug 30;6:32090. doi: 10.1038/srep32090 (PMC5004194; doi:10.1038/srep32090)
Supplement: Supplementary Information [file srep32090-s1.pdf]

SUPPLEMENTARY INFORMATION

Expression and Functional Analysis of a Novel Group of Legume-specific WRKY and Exo70 Protein Variants from Soybean

Ze Wang[1], Panfeng Li[1], Yan Yang[1], Yingjun Chi[1], Baofang Fan[2] and Zhixiang Chen[1, 2,*]

[1]Department of Horticulture, Zijingang Campus, 866 Yuhangtang Road, Zhejiang University, Hangzhou, 310058, China

[2]Department of Botany and Plant Pathology, 915 W. State Street, Purdue University, West Lafayette, IN 47907, USA

*Corresponding author (Email: zhixiang@purdue.edu)

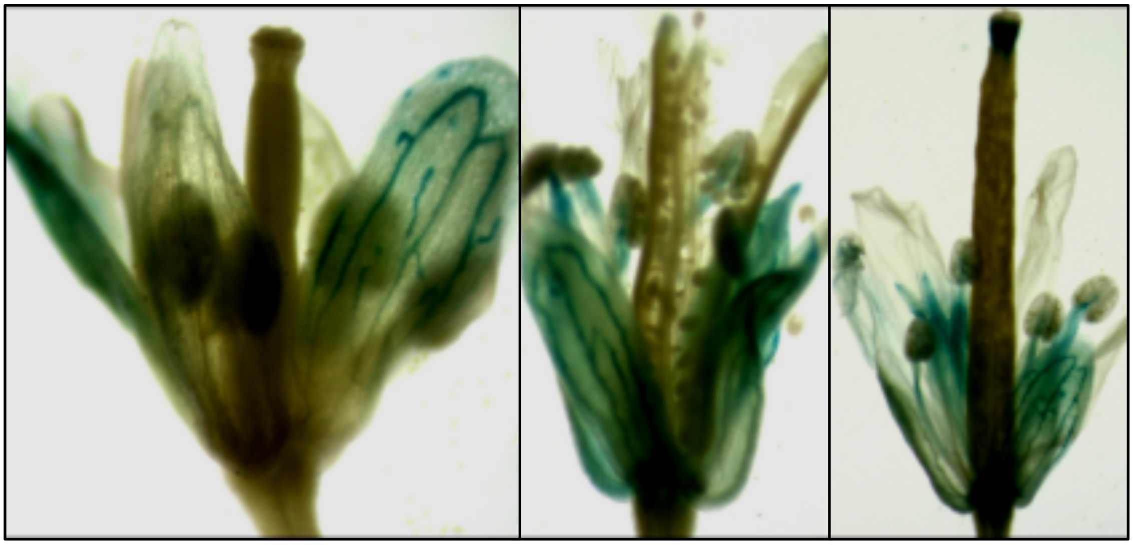

Supplemental Figure S1. Strong promoter activity of GmExo70J7 in the vascular system of sepals in transgenic Arabidopsis flowers.

Histochemical analysis of the promoter activity of *GmExo70J7* (J7) in the flowers of transgenic Arabidopsis flowers at stages 15 and 16.

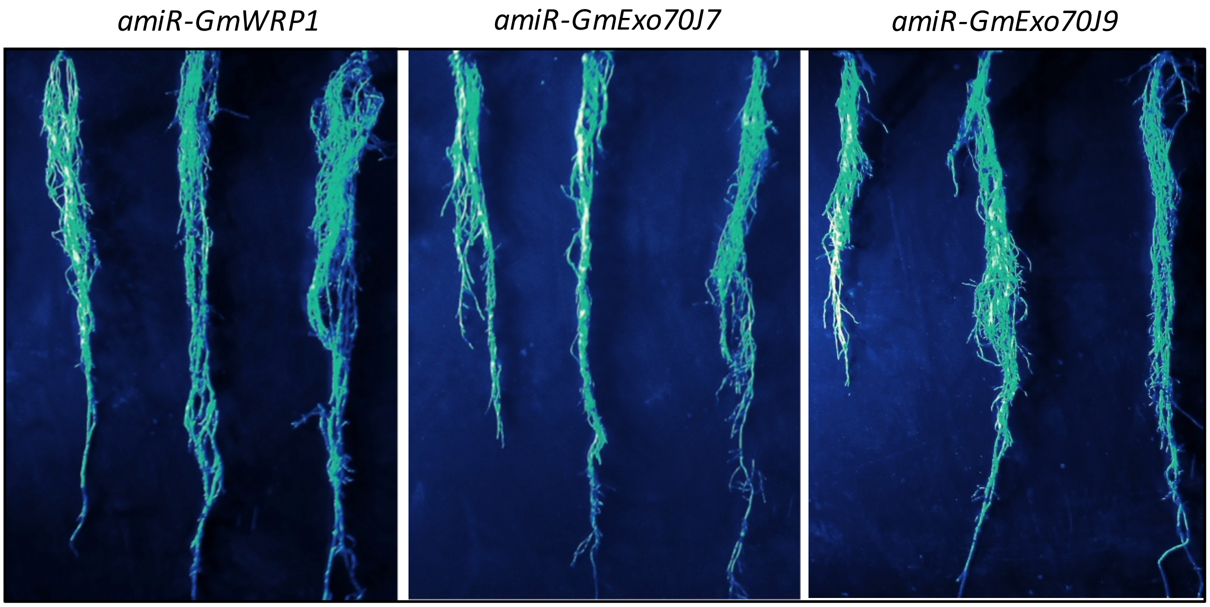

Supplemental Figure S2. Transgenic GFP-positive hairy roots containing pSM103 derivative.

Soybean hairy roots were generated after inoculation with *A. rhizogenes* cells containing pSM103 derivatives carrying amiRNAs for *GmWRP1* (*amiR-GmWRP1*), *GmExo70J7* (*amiR-GmExo70J7*), and *GmExo70J9* (*amiR-GmExo70J9*). Transgenic hairy roots were identified by their GFP fluorescence from expression in plant cells of an intron-containing GFP gene in the pSM103 binary vector. The composite soybeans with GFP-positive hairy roots were inoculated with rhizobia and the numbers of nodules was determined 35 days post inoculation.

Supplemental Table 1. Primers for generation of promoter-GUS constructs

Gene name	Gene identifier	Primers
GmExo70J1	Glyma02g39771	F: AGCAAGCTTATTAGTTTGAGCGTGGTTTA<br>R: AGCGGATCCGAGCACCGTGTTCTTTCTCA
GmExo70J2	Glyma02g39780	F: AGCAAGCTTACTATTTCTCTTAGTTACAT<br>R: AGCGGATCCGAGTTGTACTATTAGAACAC
GmExo70J3	Glyma06g21596	F: AGCGGATCCCATGTTCCTACGACGTTCAA<br>R: AGCACTAGTGAACTGTGTCACAATACTATT
GmExo70J4	Glyma06g21608	F: AGCAAGCTTCAATAACCACGACCTCAC<br>R: AGCCCATGGGATAGCCACCACCAGTTT
GmExo70J5	Glyma06g21620	F: AGCAAGCTTGATTGCTTCCTACATTTT<br>R: AGCGGATCCGAACTGTGTCACAATACTAT
GmExo70J6	Glyma06g21695	F: AGCAAGCTTCTTTCTTAGTTCAGGTCCAA<br>R: AGCCCATGGTGATAGCCACCACCAGTT
GmExo70J7	Glyma06g21710	F: AGCAAGCTTCATGCCCACAATTTAACATCTCA<br>R: AGCACTAGTGCTCTTTCTCAAGCTAGCAAAGTT
GmExo70J8	Glyma14g37750	F: AGCGGATCCCAGAACCTGTAGCCACTCCA<br>R: AGCCCATGGAGGGAGACAAGGGTTGTGATGAT
GmExo70J9	Glyma14g37763	F: AGCGGATCCCTTTTTGTTTACTGTTAAGG<br>R: AGCCCATGGTTTGGAAGTGAGAAACCCTT
GmExo70J10	Glyma14g37890	F: AGCACTAGTAGGGACAGACACCACGAC<br>R: AGCACTAGTGTTTTATCTGAAATGGGAAA
GmExo70J11	Glyma14g37920	F: AGCAAGCTTAATATCCTGGGATGGCTTTA<br>R: AGCACTAGTGTTGGTGAGATTCGAATTGC
GmExo70J12	Glyma17g35871	F: AGCGGATCCACTAAATTTGGAGCCTCAAGGTCA<br>R: AGCCCATGGGGGCACTGTGTAATGTGTTCT

Supplemental Table 2. Primers for generation of VIGS and amiRNA constructs

Construct type	Gene name	Gene identifier	Primers
VIGS	GmWRP1	Glyma14g37960	F:AAGGGATCCTCTATTGTTGCAGCCATATTTTG<br>R:TTGGGTACCTCTTCACATAGCAAGTGGGTTG
	GmExo70J7	Glyma06g21710	F:AAGGGATCCAGAAACAGAACCAGTCCT<br>R:TTGGGTACCCCATGTTGAGCTCTTCCAAT
	GmExo70J8	Glyma14g37750	F:AAGGGATCCGGAAAATCTTCATTTTCTGA<br>R:TTGGGTACCCGTTGAACAAATTGAGCTTAT
amiRNA	GmWRP1	Glyma14g37960	I:GATGTAACTTAACTGGTTTGCTCTCTCTCTTTTGTATTCC<br>II:GAGAGCAAACCAGTTAAGTTACATCAAAGAGAATCAATGA<br>III:GAGAACAAACCAGTTTAGTTACTTCACAGGTCGTGATATG<br>IV:GAAGTAACTAAACTGGTTTGTTCTCTACATATATATTCCT
	GmExo70J7	Glyma06g21710	I:GATAATCCGATAATTCGTGCCGCTCTCTCTTTTGTATTCC<br>II:GAGCGGCACGAATTATCGGATTATCAAAGAGAATCAATGA<br>III:GAGCAGCACGAATTAACGGATTTTCACAGGTCGTGATATG<br>IV:GAAAATCCGTTAATTCGTGCTGCTCTACATATATATTCCT
	GmExo70J9	Glyma14g37763	I:GATTAACCAACGTTTAATCTCCCTCTCTCTTTTGTATTCC<br>II:GAGGGAGATTAAACGTTGGTTAATCAAAGAGAATCAATGA<br>III:GAGGAAGATTAAACGATGGTTATTCACAGGTCGTGATATG<br>IV:GAATAACCATCGTTTAATCTTCCTCTACATATATATTCCT
	amiRNA-A		AGCCTGCAGCCCCAAACACAC
	amiRNA-B		AGCGGATCCCCCCATGGCGAT

Supplemental Table 3. Primers for qRT-PCR

Gene name	Gene identifier	Primers
GmWRP1	Glyma14g37960	F: ACAAATGCACACAACCCACT<br>R: AGGTGACAGGCTCTGAAGGT
GmExo70J1	Glyma02g39771	F: AGCCAGAATAGCAAGGCTGT<br>R: TAGCCAATAGGTCCCAATCC
GmExo70J4	Glyma06g21608	F: GTCTTGTCCAGACTCCAGTG<br>R: TCCTCCTTTAACCCTGCTGT
GmExo70J6	Glyma06g21695	F: GTCTTGTCCAGACTCCAGTG<br>R: TCCTCCTTTAACCCTGCTGT
GmExo70J7	Glyma06g21710	F: TCCTTCAATGAACACCTGGA<br>R: CTGCAATCTCGCAATGAAGT
GmExo70J8	Glyma14g37750	F: GGACGGTATCTTGCTCGAAG<br>R: TTGCTTGCTGGGTAATCAGA
GmExo70J9	Glyma14g37763	F: GTGGAGGGCTTCATCTGATT<br>R: AGTCGTCTCTGGACTTGGCT
GmExo70J10	Glyma14g37890	F: GTGAGCGACGACTCTGTGATA<br>R: AAATGTCCCTGCTTGCTTCT
GmExo70J12	Glyma17g35871	F: TCCCTCTTGAATTCCCAGTC<br>R: TGGTCCTCGTTGACAACATT
